# Using the Stay Strong App for the Well-being of Indigenous Australian Prisoners: Feasibility Study

**DOI:** 10.2196/32157

**Published:** 2022-04-08

**Authors:** Elke Perdacher, David Kavanagh, Jeanie Sheffield, Karyn Healy, Penny Dale, Edward Heffernan

**Affiliations:** 1 Queensland Forensic Mental Health Service Brisbane Australia; 2 Forensic Mental Health Group Queensland Centre for Mental Health Research Brisbane Australia; 3 School of Psychology The University of Queensland Brisbane Australia; 4 Centre for Children's Health Research and School of Psychology & Counselling Queensland University of Technology Brisbane Australia; 5 QIMR Berghofer Medical Research Institute Brisbane Australia; 6 Faculty of Medicine The University of Queensland Brisbane Australia

**Keywords:** First Nation, Indigenous, digital mental health, e-mental health, mental health, social and emotional well-being, SEWB, prisoner, prison, mobile phone

## Abstract

**Background:**

The gap between mental health needs and resources for Aboriginal and Torres Strait Islander people, the Indigenous people of Australia, is most marked in the prison population. Indigenous people are overrepresented in Australian prisons. In prison, this group experiences mental disorders to a greater degree than non-Indigenous prisoners. This group has also been found to experience mental disorder at a higher rate than Indigenous people in the community. In addition to pre-existing determinants of poor mental health, these high prevalence rates may reflect poor engagement in mainstream interventions or the efficacy of available interventions. In community populations, the use of digital mental health resources may help to increase access to well-being support. However, culturally appropriate digital tools have not been available to Indigenous people in prisons. The absence of feasibility and efficacy studies of these tools needs to be addressed.

**Objective:**

The aim of this study is to determine the feasibility of the Stay Strong app as a digital well-being and mental health tool for use by Indigenous people in prison.

**Methods:**

Dual government agency (health and corrective services) precondition requirements of implementation were identified and resolved. This was essential given that the Stay Strong app was to be delivered by an external health agency to Indigenous prisoners. Then, acceptability at a practice level was tested using postuse qualitative interviews with clients and practitioners of the Indigenous Mental Health Intervention Program. All Indigenous Mental Health Intervention Program practitioners (10/37, 27%) and client participants who had completed their second follow-up (review of the Stay Strong app; 27/37, 73%) during the study period were invited to participate.

**Results:**

Owing to the innovative nature of this project, identifying and resolving the precondition requirements of implementation was challenging but provided support for the implementation of the app in practice. Acceptability of the app by clients and practitioners at a practice level was demonstrated, with nine themes emerging across the interviews: satisfaction with the current Stay Strong app, supported client goal setting, increased client self-insight, improved client empowerment, cultural appropriateness, enhanced engagement, ease of use, problems with using an Android emulator, and recommendations to improve personalization.

**Conclusions:**

The Stay Strong Custody Project is a pioneering example of digital mental health tools being implemented within Australian prisons. Using the app within high-security prison settings was found to be feasible at both strategic and practice levels. Feedback from both clients and practitioners supported the use of the app as a culturally safe digital mental health and well-being tool for Aboriginal and Torres Strait Islander people in prison.

## Introduction

### Background

Mental disorders are a leading cause of the nonfatal global burden of disease. Commonly accepted estimates are that 21.2% of years lived with disability and 7.1% of disability-adjusted life years are experienced by people with mental disorders, but some researchers argue that these figures underestimate the true impact [1]. However, the picture is even more bleak for Indigenous people. Indigenous populations in western nations such as Canada, the United States, New Zealand, and Australia are particularly impacted as they have higher rates of mental disorder, substance misuse, and suicide than their non-Indigenous population [2-10]. The rates of incarceration are also higher for Indigenous people than for non-Indigenous populations, with this cohort shown to have a particularly high prevalence of mental disorders [11,12].

Aboriginal and Torres Strait Islander people, the Indigenous people of Australia, experience a burden of disease 2.3 times more than that experienced by the non-Indigenous Australian population, with mental disorders and substance use disorders accounting for 19% of this total disease burden [13]. Compared with non-Indigenous Australians, Indigenous Australians have higher rates of suicide (2 times) [13], higher rates of hospitalization for self-harm (2.7 times) [14], higher levels of psychological distress (2.6 times) [14], and higher rates of depression and anxiety [15] and are overrepresented in the mental health system (3 times) [16]. Differences in prevalence rates between Australia’s Indigenous and non-Indigenous populations suggest an increased presence of determinants of poor mental health and well-being, a lower rate of engagement in interventions, and a reduced efficacy of the interventions available for Indigenous people in Australia. Indigenous Australians are 14 times more likely to be incarcerated than non-Indigenous Australians [17,18]. Although Indigenous people represent 3.3% of the Australian population, they constitute 29% of all prisoners [17,18]. As a breakdown of the overall prison population, Indigenous women constitute 36% of the female prisoner population and Indigenous men constitute 28.9% of the male prisoner population [17,18].

The gap between mental health and well-being needs and resources is at its most marked for Indigenous people in prison [19]. It is estimated that in Australia, the 12-month prevalence of mental disorders in Indigenous women in prison is 86% and in Indigenous men in prison is 73% (including substance use disorders). This is very high compared with the 12-month prevalence of mental disorders in the general Australian population, which is 22% for women and 18% for men [12,20]. This discrepancy is evident in other western colonized countries such as New Zealand, the United States, and Canada [19], where Indigenous people experience poor mental health [2,4,9], increased risk of substance misuse [2-4], higher rates of suicide [2,5,10], and overrepresentation in the prison population [21-23]. Although there is an improving commitment to the health and welfare of First Nations people, in these jurisdictions, there is limited Indigenous-specific well-being or mental health interventions for prisoners and limited published evaluations of these interventions [19].

The Correctional Investigator of Canada, Truth and Reconciliation Commission of Canada, and Canadian parliamentary commissions called on government to improve First Nations prisoners’ access to culturally relevant intervention [24-26]. This aim is also reflected in New Zealand’s Hōkai Rangi strategy to address the overrepresentation of First Nations people in prison [27]. Within this strategy, scaling up successful interventions, developing new Indigenous-specific interventions, and deciding how best to support their delivery have been identified as key challenges [27]. In Australia, the National Agreement on Closing the Gap aims to eliminate the gap between the health and welfare of Indigenous and non-Indigenous people [28-30]. Among the priority targets to be addressed through this agreement are reducing the rates of incarceration and improving Indigenous prisoners’ social and emotional well-being (SEWB). This may be achieved in part through access to culturally safe, Indigenous developed and delivered, evidence-based interventions [28,30].

Although it is possible to develop and deliver culturally safe well-being interventions specifically for Indigenous people in prison, there is a lack of access for prisoners to evidence-based interventions [19]. There is a need for equity-oriented evidence-based interventions that engage a population with complex needs in a restrictive prison environment. The development of innovative, culturally safe, and effective interventions that make efficient use of the finite resources available to support prisoners would address the growing gap between need and service.

As a step toward achieving this vision, since September 2015, the Stay Strong Custody Project (SSCP) has been trialing culturally safe digital mental health resources with Aboriginal and Torres Strait Islander women and men in prison. This has been achieved through the use of these tools by the Indigenous Mental Health Intervention Program (IMHIP; Queensland Health’s Forensic Mental Health Service). IMHIP is an SEWB service specifically for Indigenous people in prison, offering culturally safe early intervention and support for Indigenous people in custody and for up to 6 months after their release from prison. IMHIP’s innovative service delivery model enabled the service to become an early adopter of digital mental health assessment and intervention tools within the high-security prison environment. The SSCP adapted a collection of 5 digital mental health tools to support the assessment of and intervention with IMHIP clients in prison. Internationally, digital tools or information and communication technology (ICT) are being used to support prisoners in completing day-to-day tasks, enhancing education and vocation skills, accessing family and support, and videoconferencing as a platform for psychiatry and psychotherapy sessions. However, these ICT examples are the exception rather than the rule. Where these limited programs have been introduced in Europe, United Kingdom, Canada, and the United States, they have typically involved low-security prisons, which have fewer restrictions on internet access and ICT support [31].

Internet access is prohibited or extremely restricted within prisons in most countries to prevent breaches of security. These limits are extended to devices that have the potential for communication with contacts outside the prison, such as smartphones, tablet PCs, and laptops. Restrictions on devices and internet use place limitations on the availability of digital mental health and well-being tools to prisoners, both in Australia and in other countries.

Despite this challenge, the use of digital resources to support mental health and well-being in prison is likely to increase in the coming years, given the degree to which it is already influencing the delivery of mental health support in the general community [32-34]. As this occurs, access to digital mental health support within prisons may begin to more closely mirror the level of access available in the outside community. This extension of access will be enhanced by establishing a body of evidence supporting the feasibility and efficacy of digital mental health interventions in a prison context. A concerted effort in creating, evaluating, and raising the awareness of credible digital mental health apps, programs, and services is essential.

Capitalizing on the rapid advancement and widespread adoption of digital technologies, researchers and developers have written and tested digital interventions related to a range of mental health issues, resulting in a substantial evidence base supporting their efficacy. The most widely evaluated group of digital interventions include those that target depression and anxiety in the general population, with greater support for guided digital interventions than unguided [35-41]. However, the benefit of unguided interventions is their ease of accessibility for the general population. The evidence base for digital mental health tools and systems, guided or unguided, for Indigenous people is practically nonexistent, especially for Indigenous prisoners, which limits the generalizability of these results to this population [42].

An exception is the Stay Strong app, which is used as a guided or therapist-facilitated mental health and well-being intervention tool for Indigenous clients. The app was based on the original paper-based version of the Stay Strong Plan, which has been in use since 2007 and has demonstrated significant improvements in client well-being, life skills, and alcohol dependence [43]. An iOS (Apple) version of the Stay Strong app was developed in 2013, making it the first digital mental health app specifically for the First Nations people of Australia [44]. Both the Stay Strong Plan and the app combine motivational interviewing and problem-solving strategies within a brief, strengths-based assessment and intervention tool, developed specifically for Indigenous Australians and for promoting Indigenous cultural values and self-management. Studies have demonstrated the feasibility of the app for use with Aboriginal and Torres Strait Islander people in the community to address mental health and substance misuse issues [44,45]. The app provides a structured evidence-based process to improve engagement with clients and overcome the gap in service access for Aboriginal and Torres Strait Islander clients [44]. For this project, the app is adapted to an Android version, and only this version is used in this study.

### Objective

This study assesses the feasibility of the Stay Strong app as a digital well-being and mental health tool for use with Indigenous people in prison. This study also describes the implementation of the Stay Strong app as a digital well-being and mental health tool for use with Indigenous people across 3 high-security prisons in Queensland, Australia.

## Methods

### Overview

In this section, we first explain the setting and preparatory work required to implement this project. Then, we describe the intervention itself, including the adaptations and recruitment of participants. Finally, we describe the procedure for implementing and evaluating the intervention.

### Setting

IMHIP is an SEWB and mental health service provided in prisons by Queensland Health. The IMHIP team is led by an Indigenous project manager and has a staffing profile of 8 Indigenous health worker and practitioner positions, all filled by Aboriginal and Torres Strait Islander staff. The team includes social workers, psychologists, and other Indigenous health workers specializing in mental health and SEWB. Support is also provided to IMHIP clients, as needed, by a non-Indigenous forensic psychiatrist. The IMHIP service is provided within 3 high-security Queensland prisons (Brisbane Women’s Correctional Centre, Southern Queensland Correctional Centre, and Woodford Correctional Centre)—2 women’s prisons and 1 men’s prison. Where possible, clients are matched with IMHIP staff of the same gender, in line with recommended cultural practice. The IMHIP service is offered to Indigenous prisoners on their point of entry into one of these correctional centers, with the level of service determined by the client’s level of need.

The Brisbane Women’s Correctional Centre is a high-security women’s prison with a built capacity for 264 prisoners and current state of 276 prisoners (as of May 4, 2021). The Southern Queensland Correctional Centre is a high-security women’s prison with a built capacity for 300 prisoners and current state of 302 prisoners (as of May 4, 2021). The Woodford Correctional Centre is a high-security men’s prison with built capacity of 988 prisoners but is currently over capacity with 1426 prisoners (as of May 4, 2021), making it the largest prison in Queensland.

Differences in terminology can occur internationally when describing various security levels of prisons. In Queensland, there are three prison security levels: maximum, high, and low. High security is the most common classification provided to prisoners upon entry into the prison system, including those on remand, with 94% (9176/9752; as of May 3, 2021) of prisoners being accommodated in a high-security prison. Prisoners on a high-security classification can only be accommodated in high-security prisons. In some jurisdictions, this security classification would be referred to as maximum security. In Queensland, maximum security refers to an uncommon classification applied to no more than 38 prisoners at any point of time, with all of them isolated in individual cells. A prisoner is placed under a maximum-security order only if his behavior is considered as a threat to the safety of other prisoners and staff or the security of the prison. Prisoners under a maximum-security order are placed in maximum-security units, which are separate from the main accommodation areas. Only Woodford Correctional Centre and Brisbane Correctional Centre have maximum-security units.

### Contextual Preparation

#### Overview

To our knowledge, the SSCP is the first use of digital mental health tools in Australian prisons. The project’s innovative mode of delivery required the research team to develop new pathways and protocols for delivery in prisons, from inception to implementation. IMHIP’s ground-breaking model of service delivery presented numerous challenges that were overcome through the support, consultation, and involvement of community groups and key Indigenous stakeholders.

#### Implementation Process

Textbox 1 outlines the implementation process undertaken from conception to sustained adoption of the Stay Strong app within the IMHIP service.

**Steps in implementation of the Stay Strong app in a prison setting.**

**Step 1: Conception and initiation**

- Identification of client base, app, and context.
- Scoping potential for project with stakeholders.

**Step 2: Definition, planning, and approval**

- Development of project plan and evaluation protocol.
- Gained support from the Indigenous Mental Health Intervention Program (IMHIP), Queensland Forensic Mental Health Service, leadership team of Brisbane Women’s Correctional Centre, and Menzies School of Health Research.
- Gained approval from the health and correctional government agencies for implementation of Stay Strong app into service and for evaluation.
- Gained approval and adapted Stay Strong app to the Android custody version.
- Gained approval and adapted outcome measures from paper versions to Android apps.
- Development of feedback app (Android version).
- Development of the locked down tablet interface for project.
- Practitioners trained in Stay Strong app and in the use of the app in the context of IMHIP service.
- Tested functionality, user interface design, compatibility, performance, installation, and offsite and onsite security.

**Step 3: Launch**

- September 2015—first use of Stay Strong app by an IMHIP client (Brisbane Women’s Correctional Centre)

**Step 4: Performance and support**

- Provision of technical support, clinical supervision, support in output production (practitioner reports, client cards, and management data), and continued training for practitioners.
- Introduction of Android emulator with change to Windows-based tablet PCs.

**Step 5: Project transition into sustained adoption**

- Evaluation of Stay Strong app’s feasibility and efficacy with Indigenous prisoners.
- Management of transition of technical support from research team and tool developer to health agency.
- Continued use of the Stay Strong app as part of the formal IMHIP service delivery model.

#### Preparation for Expected Challenges

Attempting to bridge the digital divide in mental health interventions in prison involved a range of challenges: prisoners being denied access to internet, lack of access to technologies, and competing philosophies of the correctional environment (security and containment) with the external health service. For the SSCP, this required approvals and protocols for the use of digital mental health strategies and technology as part of IMHIP service delivery. This included acquiring ethics and security approvals across both correctional and health agencies, the leadership teams of the relevant prisons, the Queensland Forensic Mental Health Service, and the IMHIP team responsible for the delivery of the service. The approvals and the training of IMHIP staff and those involved in the broad care of IMHIP clients ensured engagement and support for the project. From inception and acceptance-testing to implementation, this process took approximately 18 months, with the first use of the Stay Strong app and associated project apps by IMHIP clients occurring in September 2015.

In preparation for implementation, processes and safeguards were put in place to meet the security requirements of the prisons and ensure confidentiality of client data. These included the safe and secure storage of tablet PCs within health facilities and during transport from health facilities to prison facilities. It was agreed that upon entry into the prisons, practitioners would proceed through entry checks with devices being logged against practitioners’ names in permanent entry logs for officer reference. Permanent entry logs are records of what items specific staff and visitors are permitted to bring into a prison. To obtain the approval for the study, we also had to agree that, once in the prison, practitioners would abide by the security restrictions placed upon the use of tablets, supported by the use of lockdown software on devices. This was to ensure that the digital environment met security requirements.

Given the research team’s experience in working across both agencies and within a range of correctional environments, they were able to expediate many processes: pre-empting issues of security and communication; movement of digital devices through centers; concern over prisoners’ access and use of devices; management of confidential prisoner or client information; use of audio recording apps on devices for client feedback; device charging, maintenance, and ICT support; staff and stakeholder training; stakeholder and leadership engagement and approval; client’s willingness to consent to providing feedback and having their interviews audio recorded; and logistics of printing and sharing of the prisoners’ Stay Strong app client cards with prisoners and their prisoner property for use upon release.

The software developer, principal researcher, and IMHIP team completed the key elements to support adaptation and implementation: functionality, user interface design (walk-through of general design heuristics), compatibility (operating system device, screen, and plan for no connectivity), performance (memory and battery), installation, and security testing (data flow with broad agency security requirements).

Apps were password-protected to ensure client confidentiality; practitioners had to re-enter passwords to wake devices from sleep mode or to reopen the app and when accessing existing data. Tablets were also controlled using lockdown software (SureLock software; 42Gears Kiosk lockdown software for Android devices) to meet the security and confidentiality requirements of the health and correctional agencies responsible for the care of IMHIP clients.

### Project Support

Financial support for the purchase of technology, development of software, and ICT support was critical to the project’s success (refer to the *Acknowledgments* section). Throughout this process, it was essential that partnerships across health, correctional, academic, and software development sectors were developed and maintained. Without these relationships, the project would not have developed and formed a precedent for digital mental health strategies in Australian prisons.

### Intervention

**Figure 1:**
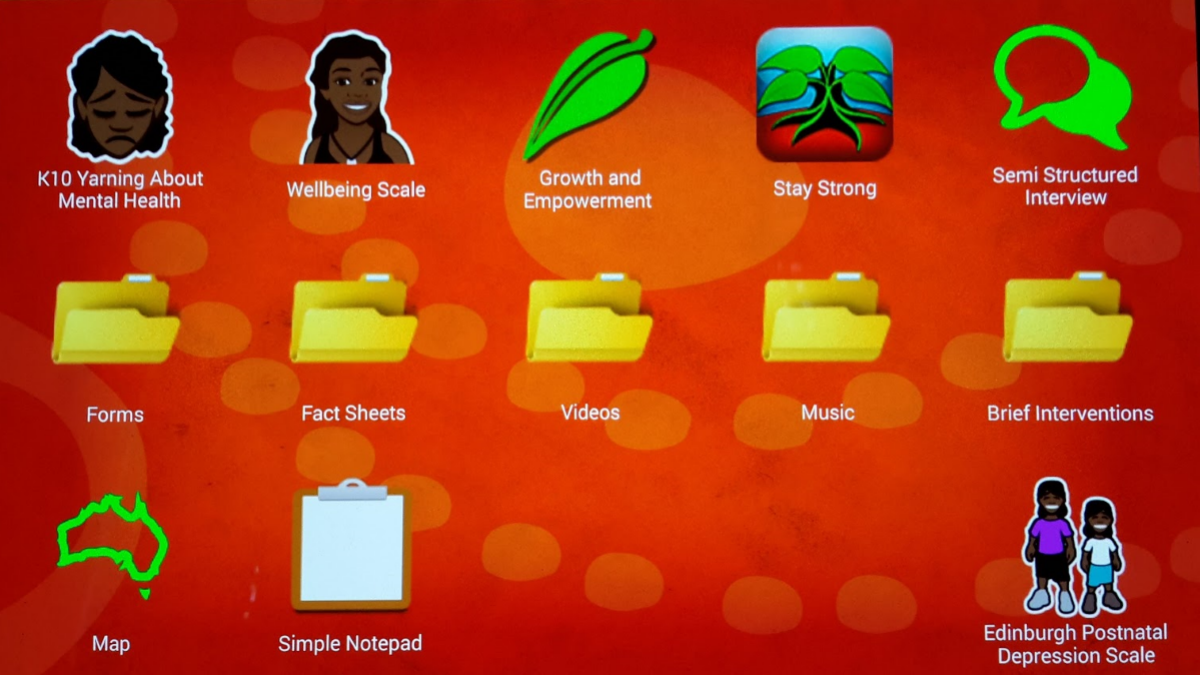
Home screen of the tablet PC showing the Stay Strong app and other related apps.

The Stay Strong app and 4 other apps adapted or developed specifically for this project provide a collection of digital mental health tools to support IMHIP clients. The Kessler Psychological Distress Scale [46], the Warwick-Edinburgh Mental Well-being Scale [47], and the Growth and Empowerment Measure [48] were used as outcome measures along with the semistructured interview app to inform the intervention and support the subsequent efficacy evaluation of the Stay Strong app. All use of digital resources was through tablet PCs, facilitated by the practitioners and used as an extension of their assessment and intervention resources (Figure 1). All the outcome measures, semistructured interview app, and the Stay Strong app were completely stand-alone apps owing to the internet connectivity restrictions placed upon the project. Consequently, all the apps used in this project were able to work offline (ie, not requiring network connection to function), thereby meeting both the security requirements of the prisons and the client confidentiality requirements of the health service.

Approval was given by the original developers of the Stay Strong app to adapt the original iOS community version to an Android custody version. Subsequently, the community version has been superseded by a hybrid version (for use on both iOS and Android devices). Similar to the community version, the steps of the custodial adaptation are ordered to enhance engagement while using a holistic approach for assessing a client’s well-being and mental health needs. This 11-step app aims to engage clients and provide a nonthreatening approach to the discussion of their well-being and mental health. A comparison between the stepped intervention process of the custodial version and the recently updated community hybrid version is presented in Multimedia Appendix 1. The stepped intervention process of both versions includes the following: (1) collecting demographics, (2) talking about family, (3) talking about a client’s strengths, (4) identifying a client’s worries, (5) setting first goal for change, (6) setting second goal for change, (7) tips to enhance emotional and physical well-being, and (8) tips to manage substance misuse (Multimedia Appendix 1).

**Figure 2:**
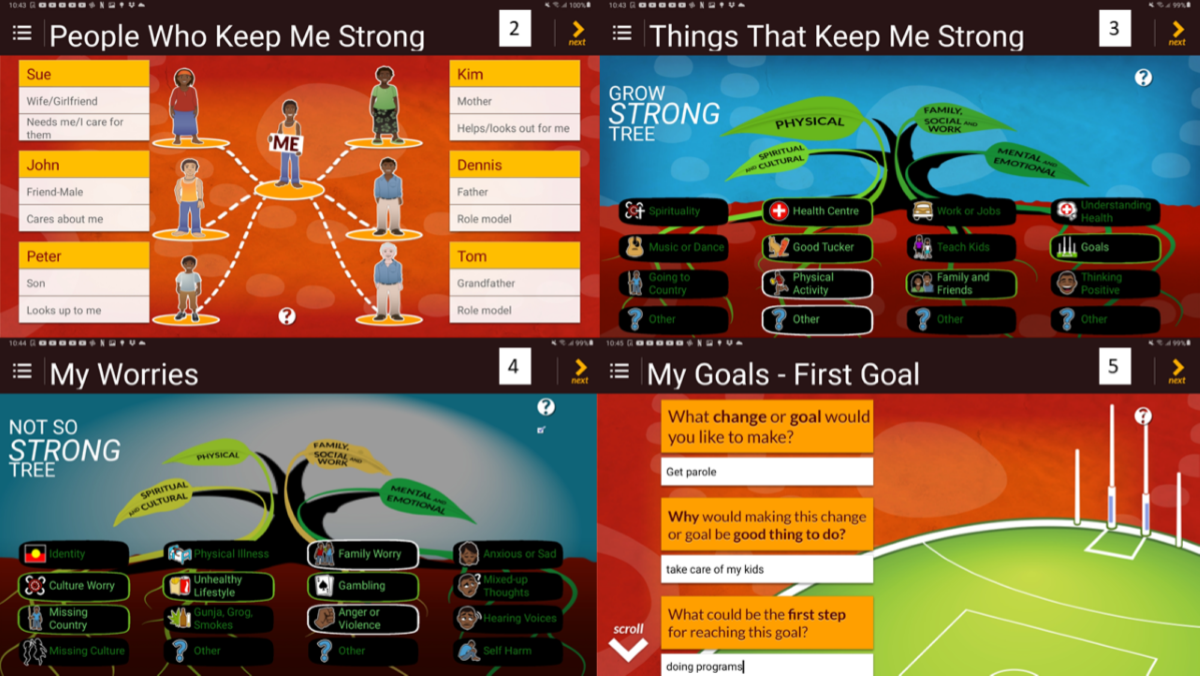
Custodial version of the Stay Strong app—steps 2-5.

**Figure 3:**
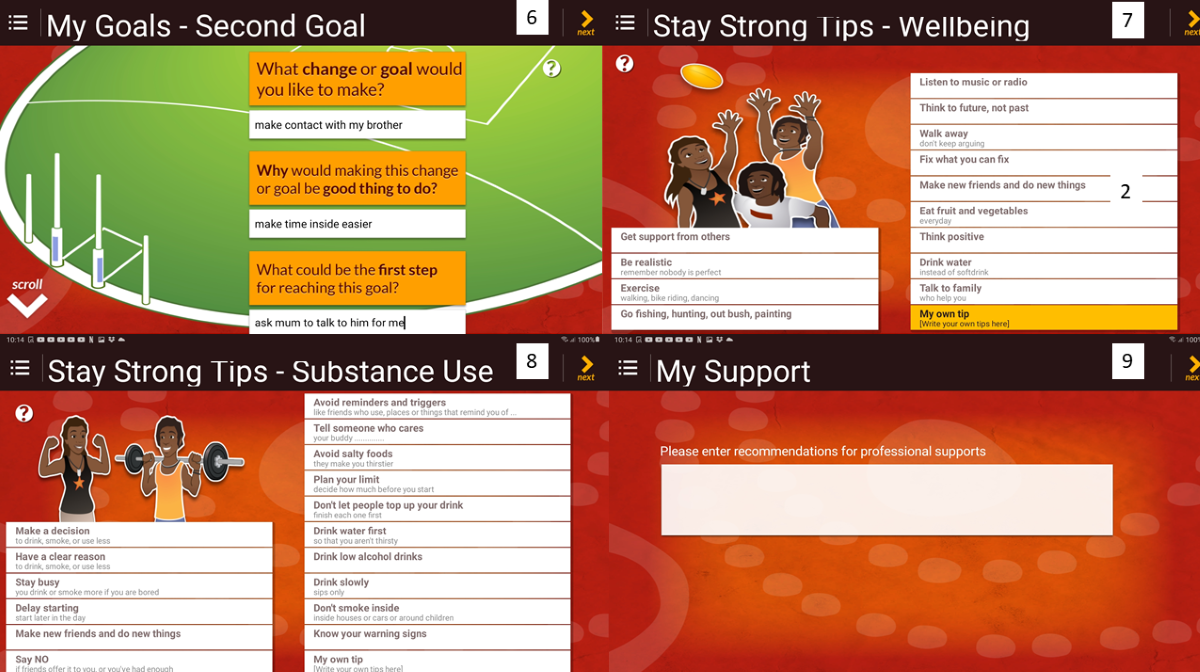
Custodial version of the Stay Strong app—steps 6-9.

Key differences in the custody version when compared with the community version of the Stay Strong app are the removal of the client photo option, research *collect information* tick box option on the demographics page, and email option on the completion page of the Stay Strong app. Removal of these options from the community version was needed to meet the client confidentiality requirements of both the health and correctional agencies for implementation of this project in 2015. Figures 2 and 3 show steps 2 to 9 of the custodial version, which is nearly identical to the community version, with the exception of the additional *my support* page.

In the custodial version of the Stay Strong app, step 9 is the *my support* page in which discussion with clients occurs regarding professional supports that may be of benefit during custody and upon release. This is important, given the risks and challenges experienced by people during their first 6 months after release from prison [49,50]. Contact details are listed in this section, which are then automatically copied to the client’s summary (step 10) and client card (step 11).

**Figure 4:**
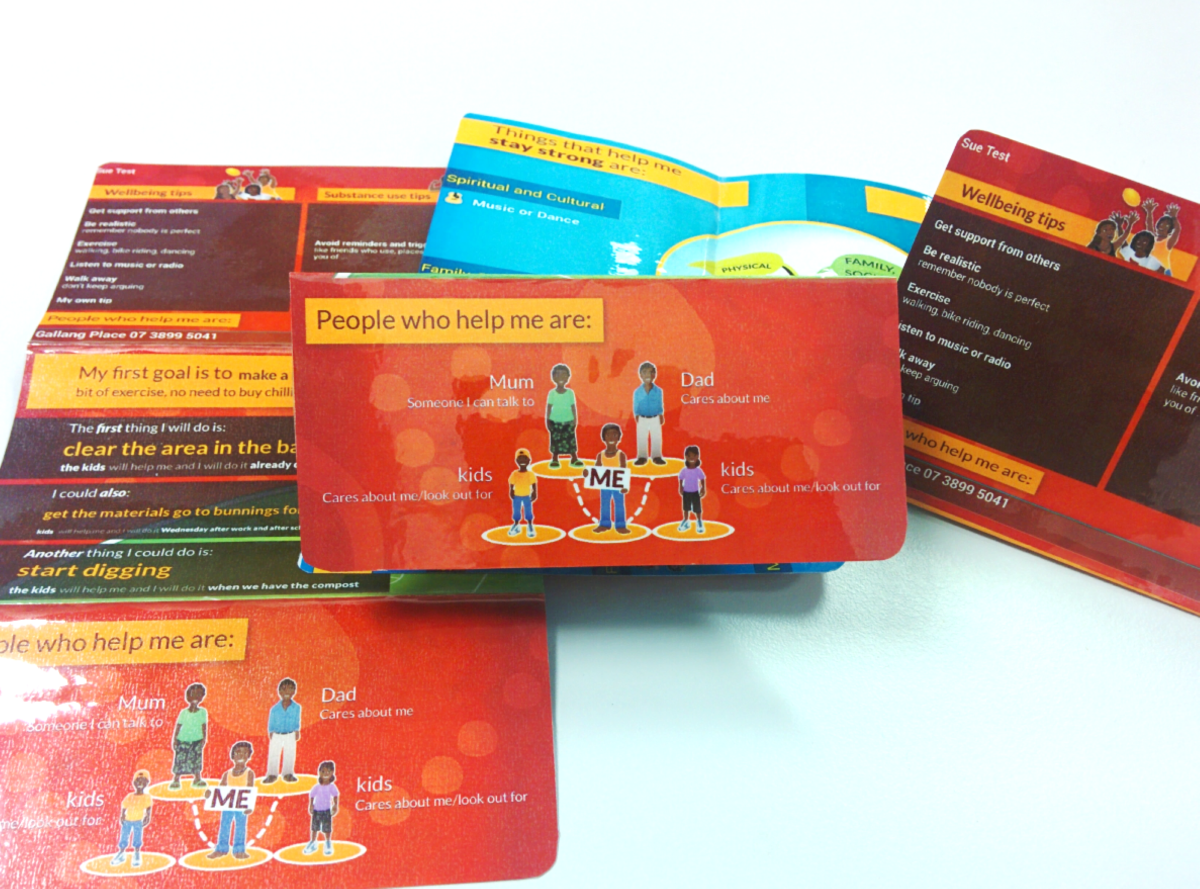
Custodial version of the Stay Strong app—Stay Strong client card (step 11).

The client summary is a pictorial summary of each step, which then can be made into the client card (Figure 4), which is a wallet-sized, folded, and laminated card version of the client summary. Clients were provided with 2 copies of their cards, one for use while in custody and another that was stored with other personal property for use upon release. The cards were also folded in a way that allowed prisoners to display the pictorial representation of their support network easily in their prison cells.

### Recruitment

#### Client Participants

Participants were recruited from the client base of IMHIP between January 2017 and September 2019 (19/27, 70% women and 8/27, 30% men). All IMHIP clients were from one of the 3 high-security adult Queensland prisons (2 women’s prisons and 1 men’s prison). The IMHIP staff recruited clients who had the capacity to consent to their involvement in the project and who were regarded as not presenting any safety concerns. An invitation to participate in the program involved both written and verbal explanations of the study and consent forms. Before their release from prison, only clients who completed the baseline and follow-up assessments were invited to participate in a semistructured feedback interview with an IMHIP practitioner. All IMHIP practitioners at the time of the study were involved in conducting the semistructured interviews with clients.

#### Practitioner Participants

During the same period, IMHIP practitioners were invited to participate in semistructured feedback interviews with the principal researcher (6/10, 60% women and 4/10, 40% men). Similar to that for the client participants, practitioners’ invitations involved both written and verbal explanations of the interviews and written consent was obtained.

Initial training for all practitioner participants involved either a 2-day trainer or 1-day Stay Strong app workshop. The process also involved shadowing of colleagues as part of the staff induction processes and Stay Strong app supervision by the principal researcher for the period of the study. The accredited Stay Strong app training package was provided by recognized trainers, and workshop content was summarized, for ongoing reference, in the Aboriginal and Islander Mental Health Initiative Stay Strong Planning Brief Treatment Manual [51].

### Ethics Approval

The study was approved by the Darling Downs Hospital and Health Service Human Ethics and Research Committee (clearance #HREC/14/QTDD/65), the Behavioural and Social Sciences Ethical Review Committee of the University of Queensland (clearance #2015000360), and the Queensland Corrective Services Research Committee (no clearance number provided) before the IMHIP clients and practitioners were invited to participate. Client participants did not receive any financial compensation for participation in the study. Payments were not allowed by the prisons in which the participants were accommodated.

### Procedure for Implementation and Evaluation

The study synthesized feedback from client and practitioner semistructured interviews to determine the feasibility of integrating the Stay Strong app into an SEWB and mental health service for Indigenous prisoners.

### Delivery of Intervention

Steps within the Stay Strong app were completed by clients with their trained IMHIP practitioners as part of the IMHIP service delivery model. This was facilitated and supported by practitioners in session. During the project, clients completed the Stay Strong and outcome measures apps (Kessler Psychological Distress Scale, Warwick-Edinburgh Mental Well-being Scale, and Growth and Empowerment Measure) at 3-month intervals (at intake, first follow-up, and second follow-up) if still in custody. All participants who were in custody for their second follow-up were offered semistructured feedback interviews with practitioners.

### Semistructured Interviews

Following their use of the intervention, participating clients and IMHIP staff were invited to provide feedback on the effectiveness of the Stay Strong app, its mode of delivery, and the service context for its use. The purpose of the interviews was to evaluate the use of the Stay Strong app and identify benefits and areas for improvement. The questions for the client semistructured interviews about their experience with the Stay Strong app are reported in Textbox 2, and those for practitioner interviews are reported in Textbox 3. The Stay Strong app went through a name change during this project, dropping *plan* from its title; thus, users were asked about their use of the Stay Strong Plan in reference to what is now known as the Stay Strong app (Textboxes 2 and 3).

**Stay Strong app’s client questions used during semistructured interview.**

**Questions**

- What were the best things about working on a tablet?
- What were the best things about developing your own Stay Strong Plan?
- What were the worst things about working on a tablet?
- What were the worst things about developing your own Stay Strong Plan?

**Stay Strong app’s practitioner questions used during semistructured interview.**

**Questions**

- What has it been like working with the tablets?
- What has it been like using the Stay Strong Plan with women/men?
- What would you suggest we do differently?
- How would you explain the tablets and Stay Strong Plan to other practitioners?
- How do you explain the Stay Strong Plan to the women/men you work with?

Before the interviews, of the 27 client participants, 24 (89%) clients consented to have their interviews audio recorded and the remaining 3 (11%) clients entered their responses directly into the tablets in response to the interview questions. All the (10/10, 100%) practitioners consented to having their interviews audio recorded.

### Analysis

Audio recordings of the feedback interviews were transcribed verbatim by the principal researcher (EP). Following transcription, researchers (EP and KH) used the constant comparison method [52] as a framework for conducting the thematic analysis, as used in previous qualitative analyses [53]. There were 2 iterations of the qualitative coding. Practitioner and client data were coded separately. On the first pass through the data, the 2 coders independently identified themes that arose from >1 respondent across the questions in >1 interview. Then, the themes were agreed upon by the research team and coded independently for each question and each respondent (yes=1; no=0). There was no limit on the number of themes that could be coded for each response. Data were entered directly into SPSS data sets, and direct quotes reflective of each of the themes were collated. At this stage, any theme that did not reach satisfactory interrater reliability were discussed until consensus between coders was attained. Following agreement on themes within practitioner and client data, EP compared the 2 sets of themes and identified commonalities between both practitioner and client themes; these were then checked by KH.

## Results

This section outlines both themes emerging from feedback interviews and responses to unexpected challenges experienced during the project.

### Stay Strong App Themes Emerging From Client and Practitioner Feedback Interviews

#### Overview

Interrater reliability across themes was moderate to strong (*κ* ranging from 0.71 to 1). The prominent themes identified across both client and practitioner interviews include the following: general satisfaction with the Stay Strong app in its current format; support of clients’ goal setting, self-insight, empowerment, cultural development, and engagement in intervention; ease of use of the Stay Strong app; and initial problems with using an Android emulator. These themes are discussed below and include example responses by both clients and practitioners.

#### Satisfaction With the Current Stay Strong App (General Positive Statements or No Recommended Changes)

There was clear support from both clients and practitioners who voiced their satisfaction with the Stay Strong app in its current format, providing general positive statements or specifically identifying that there was no need for any change in the app.

Responses from clients included the following:

Oh, I thought it was one of the best things ever.

Gives me some bit of hope in myself that you know.

Liked being able to tell my story and explain it.

Responses from practitioners included the following:

I would say ninety nine percent of the clients that I worked with them on the tablet have really enjoyed it. It's been something that they haven't done before. You know, it's exciting because they're allowed to touch a computer. I think they've enjoyed it.

It's amazing. It's very, it's, it's kind of fun. It's exciting. You feel like you've got this. You're like technologically advanced, it's something. You know, new and exciting, yeah, it's quite, it's user friendly, too, and I know it just kind of makes you, you look deadly and you, you know.

Then they love [sic] piece of technology that they can use, but they probably don't get to use at all while they're incarcerated, something different to do.

#### Supported Client Goal Setting

All participants welcomed the Stay Strong app’s support for clients’ in developing and learning the process of goal setting. Responses from clients included the following:

It got me thinking about goals, my goals.

To know that you have got goals and that you can believe in making your goals; and just being yourself and believe.

Working out a lot of my goals and things about myself that I didn’t know.

Responses from practitioners included the following:

I think it helps them gain an understanding of how to work through goals that probably never had that opportunity. Never had that experience so actually understanding the process and how to work through steps in a goal.

That it is a very useful tool for building that rapport with the client and helping them identify their future goals and their strengths and weaknesses and their support systems.

It's a cultural intervention tool used to establish, how do I say that? To establish, to help focus the kind of future goal settings and, and a, identify their support systems, strengths, weaknesses, future goals in a way that's not too invasive.

#### Increased Client Self-insight

The Stay Strong app was recognized by both clients and practitioners as enhancing the development of client’s self-insight and self-reflection. Responses from clients included the following:

So going through the app, it makes, it makes me realise that I’ve got a lot of good things about me and like I didn’t even think of it, you know. And just going through it makes me realise that I do have some positive.

I know how to think about things now before acting on it now, you know in positive ways.

You can actually see my progress like on the tablet, which is pretty good. I spun out a bit.

I think in the beginning, there's so much I didn't realise, how sad and how bad my mental health was...yeah. Very confronting.

Responses from practitioners included the following:

It sort of draws their attention to thinking and reflecting on those aspects in their, in their lives.

It is a very good reflective tool.

It's helpful for people that are leaving prison to sort of revisit and understand what their risks are. So that hopefully when they are released and back in the community that they can continuously be aware of those.

#### Improved Client Empowerment

There was agreement between clients and practitioners that the Stay Strong app represented a client-led and directed tool that enhanced clients’ confidence, view of self, and empowerment. Responses from clients included the following:

You know, in the long run, with those little steps in between. That you get, you know, you feel like you’re really proud of actually getting there.

Knowing that I can actually do it. You know like because I always doubt myself.

It helped me though, you know, it helped me through a couple of rough times.

Responses from practitioners included the following:

It's definitely client focused, client based. It’s their story. It's a chance for people to tell their story about, you know, the things that keep them strong, the things that take away their strength. It's a, it's a, it's an opportunity for people to start thinking about ways that they can, their path to recovery.

It’s client led, client driven.

Really helpful for people who are needing to gain empowerment in their lives and get their lives back on track and understand more deeply what their strengths are, and what their worries are, and help them inform goals and form a plan.

#### Cultural Appropriateness

Clients and practitioners alike identified the Stay Strong app as a culturally safe tool that supported clients. Responses from clients included the following:

Doing that Stay Strong Plan [referring to Stay Strong app] made me come out of my shell because I didn’t think that I was a person, not just any person, but also identifying myself as Aboriginal.

All of the support in bringing back the culture.

Helped me identify doing something spiritual. So thinking of my spirituality. It’s helping me try to connect even though it’s not like outside connections in here [prison].

Responses from practitioners included the following:

It's very culturally friendly. They, they are able to complete it fairly easily.

This is an opportunity for the Aboriginal or Torres Strait Islander person, the First Nations person to identify their story, to identify and acknowledge the network that they have that does support them in their recovery and healing.

So, this is really a document or a process that is, that gives an Aboriginal or Torres Strait Islander person an opportunity to use their own, their own ways and their own words in identifying what makes them strong. What takes away their strength and identifying their steps to recovery. What they need to do.

#### Enhanced Engagement

Both clients and practitioners identified the Stay Strong app as engaging and supportive of rapport between clients and practitioners. Responses from clients included the following:

The visual, the visual thing, as opposed to, the before counselling things, like it’s words, reports, it never, never got anywhere, it never hit home.

It’s more. You can relate to the pictures that to come up on it.

It was fast, better than paper, you can talk.

Responses from practitioners included the following:

When we spit out the cards and all that at the end of it and give it to the client, and they can see that, you know, that visual. We're very visual people.

I think that the tool actually helps with rapport building and helps them focus on that stuff and breaks the ice a bit and actually helps you, you know, break down some of the walls.

The focus of the app is to support that narrative approach, to support that talking space and that's really, really important or as we call it, the yarning space.

#### Ease of Use

Both clients and practitioners voluntarily stated that the Stay Strong app was easy to use, with some saying that it was easier than the typical paper assessment and intervention processes. Responses from clients included the following:

It’s a lot easier because again like up, working on paper would, I’ll probably find it more difficult because not being able to read or write properly and probably get really frustrating, probably would have. I find it is a lot easier on the tablet.

You don’t have to fill out paperwork that I might not understand.

Much easier to understand and to communicate my feelings.

Responses from practitioners included the following:

Before the Androids, we were doing the paper-based, so which is not as cool as doing it on a touch screen, it's actually not. Yeah, some people, some of the clients didn't mind writing into the stay strong plans [referring to the paper-based version of the plan]. You know, they keep diaries and journals and things like that. But generally speaking, completing the Stay Strong Plan on an Android tablet is very user friendly.

It's easy, you know, you know in a culturally appropriate way which is interactive.

They like the design. It's really simple, easy, it's easy to use.

#### Problems With Using Android Emulator

When the project moved from Android tablets to Windows-based tablets, it required the use of an Android emulator software program on the new devices. The emulator software allowed the app to run on a Windows-based device. A few problems were associated with the use of the emulator: interaction with the health agency computer system, lack of technical agency support for emulator use, and inclusion of additional steps to facilitate the download of client material. Feedback from both clients and practitioners supported the return to the earlier modes, with devices being matched to the app (Android app on Android tablet PC). Responses from clients included the following:

Losing all of the information.

Saving and crashing.

Responses from practitioners included the following:

The old ones we didn't have a problem with [referring to original Android tablets]. So simple. There was never an issue with them.

Wouldn't need to log into that second tablet thing, emulator.

It's written for an Android then maybe we could just get fast track it to using Androids. I think it will resolve a lot of issues with updates and things on that.

### Other Prominent Themes From Practitioner Interviews

#### Recommendations to Improve Personalization

Although no clients provided recommendations to improve personalization of avatars within the Stay Strong app, practitioners recommended being able to alter the client’s avatar, add pets to client’s support network, and further change the clothing of all avatars. Only the custody version of the Stay Strong app uses an avatar to represent the client, as images of prisoners are not permitted owing to prison security requirements.

Responses from practitioners included the following:

Some of them, you know, they will have a giggle when they’re clicking on the cartoon pictures to around, you know, their mum or sister. They like, you know, my brother here looks kind of like this. You know, it's, it's, it's, they enjoy that and it's quite...and it's personal. And they've chosen that themselves. But yeah, it would be cool to do the middle picture. And then they could choose their pet dog or cat or something like that. That would be kind of cool as well. Yeah. Yeah.

I'd probably make it more interactive so they could actually, you know, pick the cap they put on the child's head or do the hairstyle, you know.

I would love to see pets being added to, you know, the support, the support section. I think that would be really cool. Also, the ability to change the centre picture, but that might just be on my own.

#### Responses to Unexpected Challenges

There were several challenges that were not apparent at the inception of the project. Limitations were placed on the type of devices the project could purchase through the health agency, which initially conflicted with the security requirements of the correctional system. There were costs associated with in-agency information technology support for devices and limitations placed upon initial devices’ access to secure Wi-Fi, requiring practitioners to manually connect tablets to their workstation PCs to download outputs. Workplace health and safety requirements conflicted with secure storage requirements and charging of tablet PCs. There were issues with staff retention in the IMHIP service, which was seeking permanent funding at the time of the project.

As the project progressed, prison officials recognized the effectiveness of safeguards and protocols. Then, this allowed the IMHIP service to move from locked down Android tablets to Windows-based tablets that had access to the secure Queensland Health network within prison facilities. This change in device capability and access to secure networks via Wi-Fi allowed the new tablets to be used by practitioners as mobile workstations within and outside the prisons. Thus, although the restrictions for use remained the same for prisoners during appointments, practitioners’ use of devices between sessions expanded, enhancing the administration efficiencies for staff. Although overall this was a positive outcome, there were issues in gaining ICT support for the app emulator software needed for continued use of the new Windows-based tablets.

## Discussion

### Principal Findings

To our knowledge, this paper describes the first project to successfully implement the use of digital mental health apps in prisons in Australia, with its use by the first client occurring in September 2015. The description and evaluation of this project through this paper provides an example to other jurisdictions that seek to implement digital mental health solutions within the prison environment; it also provides evidence of acceptance and engagement by First Nations prisoners with a culturally safe digital mental health app.

The Non-adoption, Abandonment, Scale-up, Spread, and Sustainability [54] domains provide a framework to understand and reflect on the implementation experience and participant feedback. Below, we use this framework to discuss challenges and successes and make recommendations for the long-term sustainability of the Stay Strong app in prison.

A long-identified consideration for successful implementation in the digital health domain is the relationship between users (clients, practitioners, and developers), technology (including design), and organizations or wider systems with their economic realities [54,55]. For this project, a shared vision was held by practitioners, management, research team, and agencies that focused on improving the mental health and well-being of Indigenous prisoners. Although the correctional agency and leadership within the forensic mental health service allowed for innovation in the adoption of the app at a service level, there was tension between this and the wider health agency support for technology change. The health district only supported iOS apps, and therefore, to overcome issues of software incompatibility, the Android emulator was used, which was itself problematic at times. Although the custody version of the app was developed without funding, further development in a hybrid format (for use on both iOS and Android devices) may offer a more stable and valued proposition for funders. With development of the new hybrid version of the app and precedents set in other health districts for use on Android devices, it is hoped that these issues are less likely to pose barriers to dissemination and upscaling, if the IMHIP service expanded. Thus, it is evident that development and implementation would have benefited from cross-disciplinary involvement being extended to ICT policy makers from the beginning of the project.

The new hybrid version of the app allows for greater flexibility in use. However, given the restrictions placed upon the use of technology within high-security prisons, there is still a need for a custody version. Therefore, without customization of the hybrid or iOS Stay Strong app, Android is the only operating system for which there is a custody version. Ultimately, the value of the app is defined by its clinical relevance to users—its health benefits and financial viability [56]. Although client and practitioner feedback confirmed that the Stay Strong app’s design aims to promote client well-being, the financial investment needed to overcome the innovation-system conflict remains a hindrance to the sustained adoption in the Australian custodial environment.

Feedback through interviews revealed that the visual appeal, interactive interface, and ease of use supported clients’ engagement. Although client engagement is the natural focus of app development, practitioner acceptance has been demonstrated to be the strongest determinant of health technology adoption [54]. Peer support for the appropriateness of the technology and credibility of the tool is key to practitioner acceptance [54,57]. This project has been an example of peer influence supporting sustained adoption of digital technologies through training (including the onboarding of new staff), acknowledgment of the tool’s credibility by a national index of Australian evidence-based digital mental health resources [58]), support from the management and practitioners, and inclusion of the tool into the formal IMHIP service delivery model. Typical reasons for practitioners’ resistance to new health technology include implementation requiring policy change, inefficiency of the technology, risk of compromised practice, and negative change to client relationships [54,59]. By ensuring that these key areas were addressed, the likelihood of sustained adoption of the app was increased.

To protect against the risk of compromised practice, the knowledge domain was another focus for sustained adoption. This domain extends beyond practitioners’ and clients’ direct knowledge of how to use the app. The broader knowledge required for its use involved understanding how best to facilitate its adoption. For the SSCP, and therefore IMHIP, this has required understanding of the app’s use in clinical practice within a context involving both health and correctional agencies. This involved understanding the technical requirements of use and an ability to provide and receive clinical supervision. Deficits relating to an implementation facilitator’s broader knowledge of the service, technical support, organizational readiness, and ability to provide ongoing supervision have been identified as reasons for poor adoption rates after training by other services [60,61]. In contrast, one of the most important driving factors for this project’s sustained implementation has been the support and direction provided by its internal facilitators. The SSCP is an example of a project that involved continued training from developers, technical support from project software developers, supervision and project management by the principal researcher, and support in service delivery from the IMHIP service and forensic mental health service management. The benefit of implementing this digital mental health tool within a small developing service is the flexibility within the service delivery model to determine the most efficacious use and the support available from internal facilitators. The successful implementation and use of the app by IMHIP for >5 years at the time this paper was written demonstrates the feasibility of its use with Indigenous people in prison and, more broadly, the implementation of digital mental health tools within the prison environment. The app has evolved in use and content over this period. Both clients and practitioners value its functionality, engaging appeal, cultural appropriateness, and clinical value in goal setting, insight, and empowerment.

### Limitations

The study used implementation and interview data only. To enhance sustained adoption by and value proposition for users and agencies, there is a need for additional research into the efficacy of the Stay Strong app with Indigenous people in prison. We hope to build toward this with the future publication of a pilot efficacy study.

The project was also limited to 3 sites covered by the IMHIP service; therefore, although the user population is representative of the broader Aboriginal and Torres Strait Islander population, the number of participants was limited. The fact that IMHIP itself was a temporary project at the time meant that the service periodically experienced high staff turnover and shortages. The periods of low staffing resulted in delays in intakes and follow-ups and in combination with the short periods of imprisonment for most IMHIP clients, reduced the overall number of participants who made it to their second follow-up before release. This ultimately limited the number of IMHIP clients who were able to be included in this evaluation. However, the sample size of clients who participated in this study is comparable with other qualitative studies assessing feasibility [62-64].

The other limitation to this sample was the relatively low proportion of men participating in the program and evaluation. The main reason for this was that the participants were recruited from 2 women’s prisons and 1 men’s prison. Therefore, our sample does not reflect the higher proportion of men than women in the prison population. Future research could examine barriers to and enablers for engagement in such a program from the perspective of gender.

### Conclusions and Future Directions

The aim of this study is to determine the feasibility of the Stay Strong app as a digital well-being and mental health tool for use by Indigenous people in prison. The app was successfully implemented and it provided support, as expected, for client treatment and transition planning (transitioning from prison back into the community). It offered a culturally safe tool, delivering a match between the aims of the app and clients’ needs.

Successful implementation of this digital mental health tool required the coordination of gatekeepers (management), users (practitioners and clients), and systems (agencies, environment, and technology). This innovative project demonstrated the meaningful, manageable, and sustainable adoption of digital mental health technologies into the high-security prison environment; it provides an example for other jurisdictions that are considering the implementation of digital mental health solutions for First Nations people in prison. The thematic synthesis of client and practitioner feedback on the Stay Strong app confirmed that it was culturally appropriate and helpful in developing clients’ empowerment, self-insight, and goal setting. Users described the tool as easy to use, engaging, and supportive of client disclosure.

As discussed, previous community-based implementation studies of the Stay Strong app identified the complexity of digital mental health tools needing more than training, external follow-up, and external supervision [60]. Using the Non-adoption, Abandonment, Scale-up, Spread, and Sustainability framework [54], this study identified additional factors, which supported its implementation success. Key elements of the project’s success were the match between the app and client needs, the benefits for users of app outputs, and having internal facilitators that drive the implementation and sustained adoption of the app.

Within the SSCP, the activity of supporting adoption of this app was a deliberate social process focusing on practice change through the support and training of practitioners, removal of barriers to implementation, provision of resources, monitoring of progress, and promotion of change [65]. Ultimately, the implementation steps that the project took to achieve this success provide a framework for other agencies and jurisdictions that want to implement either the app in prison or, more broadly, digital mental health tools within the prison environment. The project has demonstrated that the Stay Strong app and other digital mental health tools provide a novel and innovative opportunity for health and correctional agencies to bridge the divide in First Nations prisoners’ mental health and well-being.

## Appendix

Multimedia Appendix 1 Comparison between community and custody versions of the Stay Strong app.

## Abbreviations

ICT: information and communication technology
IMHIP: Indigenous Mental Health Intervention Program
SEWB: social and emotional well-being
SSCP: Stay Strong Custody Project
