# Supplementary material for: Using the Stay Strong App for the Well-being of Indigenous Australian Prisoners: Feasibility Study
Source: JMIR Form Res. 2022 Apr 8;6(4):e32157. doi: 10.2196/32157 (PMC9034424; doi:10.2196/32157)
Supplement: Multimedia Appendix 1 [file formative_v6i4e32157_app1.docx]

Multimedia Appendix 1 - Supplementary Table S1. Comparison between community & custody versions of the Stay Strong app

| Stay Strong app – Community Version – 2021 hybrid release | Stay Strong app - Custody Version |
| --- | --- |
| Steps | Steps |
| 1. Demographics 2. People Who Keep Me Strong 3. Things That Keep Me Strong 4. My Worries 5. My Goals – First Goal (setting goals for change) 6. My Goals – Second Goal 7. Stay Strong Tips - Wellbeing (emotional and physical) 8. Stay Strong Tips - Substance Abuse 9. Summary of Stay Strong app – copy of all pages 10. Provision of summary hardcopy to client as A4   (Links to external resources and websites) | 1. Demographics (with photo and research collect information options removed) 2. People Who Keep Me Strong 3. Things That Keep Me Strong 4. My Worries 5. My Goals – First Goal (setting goals for change) 6. My Goals – Second Goal 7. Stay Strong Tips - Wellbeing (emotional and physical) 8. Stay Strong Tips - Substance Abuse 9. My Support (open text option typically used for professional supports) 10. Summary of Stay Strong app – copy of all pages 11. Provision of summary hardcopy to client as folded card |
| Outcome Measures embedded in new Stay Strong app | SSCP Outcome Measures developed as separate SSCP apps |
| - Kessler Psychological Distress Scale - 10 (K10) - Kessler Psychological Distress Scale - 5 (K5) - Patient Health Questionnaire - 2 (PHQ-2) - Session rating questions | - Kessler Psychological Distress Scale - 10 (K10) app - Growth and Empowerment Measure (GEM) app - Warwick Edinburgh Wellbeing Scale (WEMWBS) app - Client feedback app |
| Stay Strong app Output | Stay Strong app Output |
| Email or export to device option   - HTML text summary document - PDF pictorial depiction of Stay Strong app summary - CSV – data summary of Stay Strong apps completed on device | Export to device option only (email option removed)   - HTML text summary document (used by practitioner in practitioner reports) - PDF pictorial depiction of Stay Strong app summary (used to form client card) - CSV – data summary of Stay Strong apps completed on device (used in management summary) |
| Operating system – hybrid therefore both iOS and Android | Operating system - Android |
